# Risk Predictive Model Based on Three DDR-Related Genes for Predicting Prognosis, Therapeutic Sensitivity, and Tumor Microenvironment in Hepatocellular Carcinoma

**DOI:** 10.1155/2022/4869732

**Published:** 2022-09-30

**Authors:** Renzhi Hu, Xiping Liang, Qiying Li, Yao Liu

**Affiliations:** Department of Hematology-Oncology, Chongqing University Cancer Hospital, Chongqing Key Laboratory of Translational Research for Cancer Metastasis and Individualized Treatment, Chongqing, China

## Abstract

Hepatocellular carcinoma (HCC) is the seventh most common malignancy and the second most common cause of cancer-related deaths. Tumor mutational load, genomic instability, and tumor-infiltrating lymphocytes were associated with DNA damage response and repair gene changes. The goal of this study is to estimate the chances of patients with HCC surviving their disease by constructing a DNA damage repair- (DDR-) related gene profile. The International Cancer Genome Consortium (ICGC) and The Cancer Genome Atlas (TCGA) provided us with the mRNA expression matrix as well as clinical information relevant to HCC patients. Using Cox regression and LASSO analysis, DEGs strongly related to general survival were discovered in the differentially expressed gene (DEG) study. In order to assess the model's accuracy, Kaplan-Meier (KM) and receiver operating characteristic (ROC) were used. In order to compute the immune cell infiltration score and immune associated pathway activity, a single-sample gene set enrichment analysis was performed. A three-gene signature (CDC20, TTK, and CENPA) was created using stability selection and LASSO COX regression. In comparison to the low-risk group, the prognosis for the high-risk group was surprisingly poor. In the ICGC datasets, the predictive characteristic was confirmed. A receiver operating characteristic (ROC) curve was calculated for each cohort. The risk mark for HCC patients is a reliable predictor according to multivariate Cox regression analysis. According to ssGSEA, this signature was highly correlated with the immunological state of HCC patients. There was a significant correlation between the expression levels of prognostic genes and cancer cells' susceptibility to antitumor therapies. Overall, a distinct gene profile associated with DDR was identified, and this pattern may be able to predict HCC patients' long-term survival, immune milieu, and chemotherapeutic response.

## 1. Introduction

Hepatocellular carcinoma (HCC) remains one of the most aggressive solid malignancies throughout the world, and fatty liver, alcoholic liver, and hepatitis B and C infections are the three most significant risk factors for HCC [1, 2]. The incidence of HCC is highest in underdeveloped nations, but chronic hepatitis C virus infection, which causes liver cirrhosis, is also increasing in wealthy nations [3, 4]. Researchers have been investigating the molecular pathways underlying the pathogenesis of hepatocellular carcinoma for several decades [5]. Gene mutations, epigenetic changes, and dysregulation of coding or noncoding genes were found to influence HCC growth [6, 7]. Although we have made great progresses in integrating treatment plans for HCC and our understanding of its epidemiology, etiology, biology, diagnostics, and therapy, the long-term prognosis of HCC patients remains unfavorable [8, 9]. Metastatic illness, in which tumor cells invade nearby tissues and organs and spread cancer throughout the body, is responsible for the vast majority of cancer-related deaths. Therefore, identifying molecular markers for early diagnosis, survival prediction, and recurrence monitoring of HCC is very important. In this way, patient categorization can be improved, and medical intervention can be more effective.

All biological activities result in DNA damage because DNA damage repair keeps the genome stable and intact [10]. Several chronic illnesses, including cancer, are characterized by genomic instability. The integrity of DNA is of utmost importance in this respect, as it may prevent genomic instability [11]. In spite of the relatively low frequency of DNA damage, it should be repaired as soon as possible to demonstrate the accurate transmission of genetic information [12, 13]. Inability of the DDR to repair the following endogenous and external insults would lead to (1) a future malignant transformation, (2) the emergence of cancer, and (3) further deterioration of the DNA repair system [14]. The DDR mechanism can be modified during tumor formation or during therapy-induced tumor evolution to provide tumor clones with new growth abilities when they have lost genomic integrity and are outgrowing their original hosts [15, 16]. Cancer cells may also be more resistant to chemotherapy if DDR genes are expressed differently. Ovarian and prostate cancers may benefit from therapeutic targeting of DDR-related genes [17, 18]. Numerous studies have shown that the numerous DDR gene polymorphisms together affect the chance of developing HCC [19, 20]. In the wake of immunotherapy, researchers are placing a renewed emphasis on DDR pathways, the modifications of which are associated with hereditary traits, such as elevated TMB, caused by the accumulation of certain uncorrected DNA damage [21, 22]. DDR-related genes are linked to a poor prognosis for HCC, but the evidence is limited.

Clinical data and the expressing pattern of mRNAs of HCC patients were obtained from a publicly accessible dataset. A predictive signature of differentially expressed genes associated with DDR was then created in TCGA cohorts, and its stability and dependability were tested in the ICGC cohorts. Moreover, we examined the relationship between immune infiltrates and the expressions of prognostic genes. Furthermore, we examined the relationships between prognostic genes' expressions and characteristics of cancer that make it resistant to chemotherapy. New treatment plans for HCC patients can be created based on our discoveries.

## 2. Materials and Methods

### 2.1. The Acquisition and Processing of Data

374 HCC samples and 50 nontumor samples were presented on the UCSC Xena website (https://xenabrowser.net/). The raw gene microarray expression data of International Cancer Genome Consortium (ICGC-LIRI-JP) and associated clinical information were downloaded from ICGC. Furthermore, we eliminated datasets without clinical data. An average value was assigned to genes with two or more probe matches, while probes with two or more matches were disqualified.

### 2.2. Identification of Variation in the Expression of DDR Genes in HCC

Our statistical analysis and data visualization were performed using the R programming language. A differentially expressed gene from the DDR gene sets was also analyzed using the limma program at a significance threshold of *p* less than 0.05 and a fourfold change. “Pheatmap” was used to display the heatmap graphic.

### 2.3. Identification of Survival-Related DDR Genes in HCC

A single-variate Cox analysis was used to identify survival-related DDR genes, and the Benjamini and Hochberg correction was applied to alter the *p* value. A *p* < 0.05 was considered statistically significant.

### 2.4. Creation and Validation of the DD-Related Prognostic Signature for HCC

In order to reduce the dimensionality of intersecting genes, we used a LASSO regression analysis. DDR score-related predictive risk signatures were then optimized by including both forward and backward components. According to various fitting results, we also obtained the minimal AIC value. In the end, three gene construction models were achieved: CDC20, TTK, and CENPA. There are three components to the risk score: (0.0496 × CDC20) + (0.244 × TTK) + (0.245 × CENPA). Each patient's risk score was calculated by the use of above algorithm. The performance of the prognostic risk model was evaluated between the training cohort and validation cohort by dividing patients into low- and high-risk groups based on median and ideal cut-off points. A survival study was conducted using the Kaplan-Meier method. It was determined whether the risk mark was accurate by using a ROC curve. The survival-ROC R package was utilized to assess the t-ROC prediction capability. We also assessed the relevance of each parameter to overall survival (OS) using Cox proportional hazard regression.

### 2.5. Microenvironmental and Immune Analysis of Tumors

We examined the amount of stromal and immune cell infiltration in various tumor tissues according to the stromal score and immune score. Spearman correlations were used to investigate the relationship between the risk score and those scores.

### 2.6. Chemotherapy Sensitivity Analysis

NCI-60, which contains 60 distinct cancer cell lines from 9 different cancer types, can be accessed through the CellMiner interface (https://discover.nci.nih.gov/cellminer). A Pearson correlation analysis was performed to determine whether the critical genes were related to medication sensitivity. A correlation analysis was done on 263 FDA-approved and clinically trialed medications to determine their therapeutic impact.

### 2.7. Statistical Analysis

Analysis and installation of the R packages mentioned above were performed using the R software version 3.6.3 (The R Foundation for Statistical Computing, 2020). There are two sides to every statistical test. Statistical significance was defined as a *p* value less than 0.05. The chi-square test or Fisher exact test was used for categorical variables, and the *t*-test or Wilcoxon rank-sum test was used for continuous variables. Kaplan-Meier analysis was also performed to determine OS. Log-rank tests were used to compare survival rates between subgroups. With R's “survival” package, we conducted univariate and multivariate Cox proportional hazard analyses. Hazard ratios (HR), 95% confidence intervals, and *p* values were calculated.

## 3. Results

### 3.1. Identification of Prognostic DDR-Related DEGs in HCC

**Figure 1:**
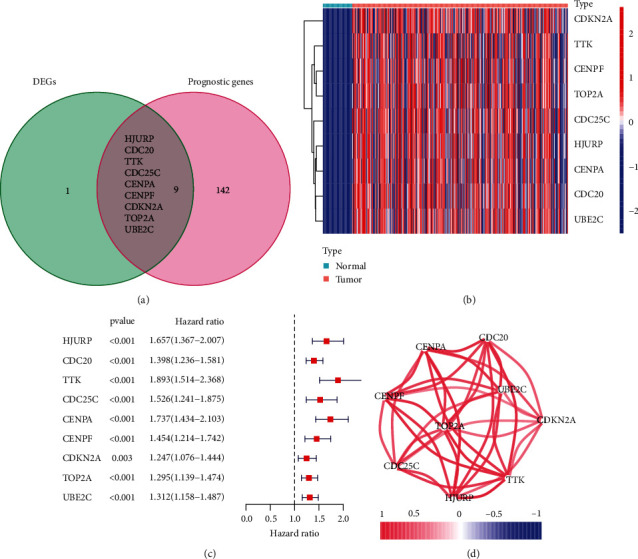
A list of possible DDR-related genes identified in the TCGA cohort. DEGs between nearby normal specimens and HCC specimens are calculated using a Venn diagram (a). (b) Expression of nine genes that overlap between neighboring normal tissues and HCC tissues. (c) Forest plots showing the associations between OS and the expression of 9 overlapping genes. (d) Correlation network of candidate genes.

TCGA datasets were used to screen dysregulated DDR-related DEGs between HCC cases and nontumor specimens. A total of ten DDR-related genes were differentially expressed between nontumorous tissues and tumorous tissues. Using a univariate Cox analysis (Figure 1(a)), a link was found between OS and 9 of them. A heatmap was used to show the expression pattern of the nine prognostic DDR-related DEGs (Figure 1(b)). As a prognostic marker, 9 DDR-related genes were kept (Figure 1(c)), and the overall risk ratio for each gene was calculated. As shown in Figure 1(d), these genes are related. In addition, we performed GO assays and found that the 151 survival-related DDR-related genes were mainly associated with regulation of cell cycle phase transition, nuclear division, chromosomal region, nuclear chromosome, ATPase activity, and damaged DNA binding (Figure S1A). Moreover, the results of KEGG assays confirmed that the 151 survival-related DDR-related genes were mainly associated with cell cycle, PI3K-AKT pathway, DNA replication, p53 signaling pathway, and platinum drug resistance (Figure S1B).

### 3.2. Development of a Prognostic Gene Signature Based on DDR-Related Genes

**Figure 2:**
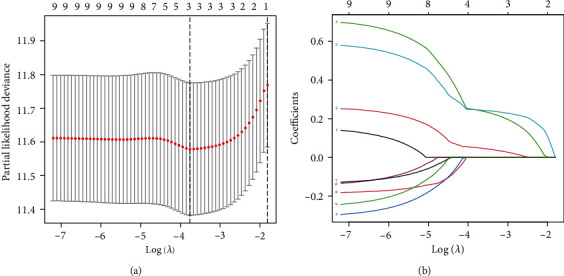
Gene signatures associated with DDR were identified in TCGA datasets using LASSO regression analysis. (a) Choosing the optimal LASSO model parameter (lambda). (b) LASSO coefficient profiles of the nine prognostic DDR genes.

**Figure 3:**
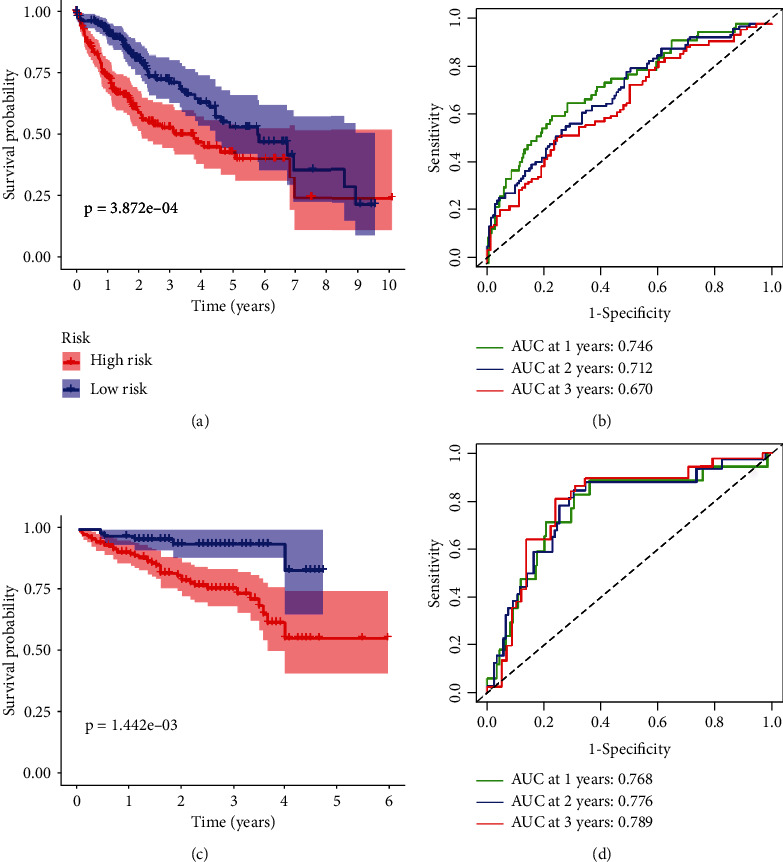
The performance of DDR-related gene signature in TCGA and ICGC datasets. Based on Kaplan-Meier analysis of the (a) TCGA and (c) ICGC datasets, patients with lower risk ratings had greater overall survival than those with higher risk scores. ROC curves were used to assess the prognostic signature's accuracy in the (b) TCGA and (d) ICGC datasets.

**Figure 4:**
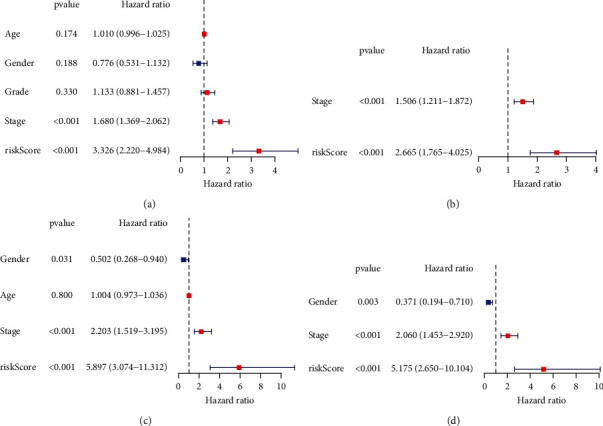
The OS by Cox regression model's univariate and multivariate evaluations. Datasets (a, b) from the TCGA. Datasets (c, d) from the ICGC.

Three DDR-related gene signatures were screened using LASSO and Cox regression analyses in order to predict OS in HCC patients from TCGA datasets: expression of CDC20 (∗0.0496), TTK (∗0.244), and CENPA (∗0.245) (Figures 2(a) and 2(b)). Patients were divided equally into low-risk and high-risk groups. Patients with low-risk marks had a greater survival rate than those with high-risk marks (*p* < 0.01, Figure 3(a)). According to time-dependent ROC analysis, the predictive precision of the DDR-related gene signature was 0.746 at 1 year, 0.712 at 2 years, and 0.670 at 3 years (Figure 3(b)). To examine the stability of the model built from the TCGA cohort, we divided the patients in the ICGC cohort into high-risk or low-risk groups based on the median value from the TCGA cohort. According to Figure 3(c), patients with a high-risk score had a shorter OS, similar to the results reported in the TCGA cohort. AUCs for the 8-gene signature were 0.768, 0.776, and 0.789 at 1, 2, and 3 years (Figure 3(d)). In univariate Cox analysis of TCGA cohorts, OS and risk markers showed a strong correlation (Figure 4(a)). After controlling for additional confounding variables, the risk score remained an independent predictor of OS (Figure 4(b)). A similar effect was also observed in the ICGC group (Figures 4(c) and 4(d)).

### 3.3. Risk Score for the Prognostic Model and Clinical Characteristics

**Figure 5:**
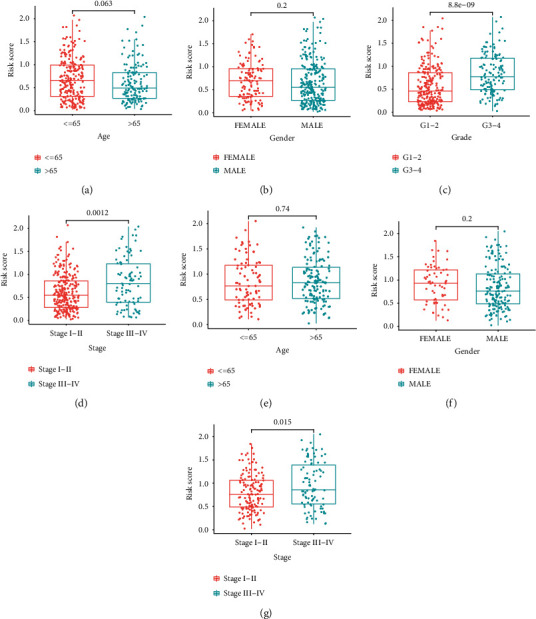
The risk score in different groups divided by clinical factors. TCGA cohort (a–d) and ICGC cohort (e, f): (a) age; (b) gender; (c) grade; (d) clinical stage; (e–g) age, gender, and clinical stage.

The relationship between risk mark and clinical features of HCC patients in the TCGA cohort revealed no relationship between age and sex (Figures 5(a) and 5(b)). In contrast, HCC specimens with advanced grade and clinical stage had a greater risk mark (Figures 5(c) and 5(d)). A similar outcome was found in the ICGC cohort as well (Figures 5(e)–5(g)).

### 3.4. Immunity and Tumor Microenvironment Analysis

**Figure 6:**
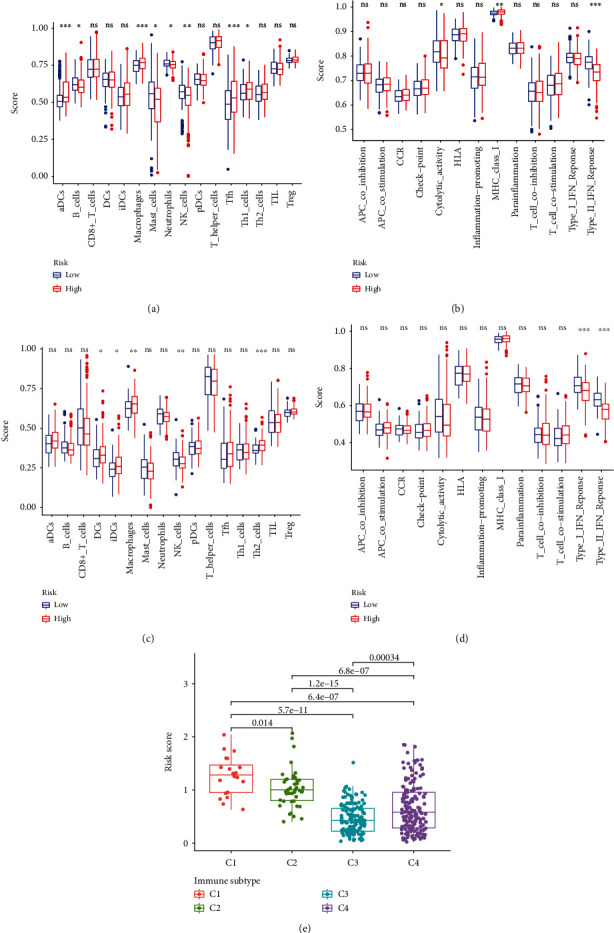
A correlation between the tumor microenvironment and risk markers. The characteristics of 16 immune cells (a, c) and 13 immune-related activities (b, d) were illustrated in boxplots. (e) Comparison of risk scores across several subtypes of immune infiltration.

To better understand the relationship between risk marks and immunological state, we measured enrichment scores of various immune cell subpopulations, functions, and pathways. High-risk groups had significantly more components of the antigen presentation pathway in the TCGA cohort, such as aDCs, macrophages, Tfh, Th1 cells, and MHC class I. (Figures 6(a) and 6(b)). Figures 6(c) and 6(d) show that the high-risk group had significantly more DCs, iDCs, macrophages, and Th2 cells in ICGC datasets. The immune infiltration types C1 (wound healing), C2 (IFN-g dominant), C3 (inflammatory), C4 (lymphocyte deficient), C5 (immunologically silent), and C6 (tumor-inhibiting) have been identified in malignancies (TGF-*β* dominant). The HCC C6 immune subtype can be classified only in one patient sample, and the C5 immune subtype cannot be classified in any patient sample. Therefore, the immunological subtypes C5 and C6 were omitted. A correlation was discovered between the two risk scores for HCC and immune infiltration, according to the TCGA-HCC data. A strong correlation was found between high-risk marks and C1 and a strong correlation between low-risk marks and C3 (Figure 6(e)).

### 3.5. The Expression of Prognostic Genes and Chemotherapy Response in Cancer Cells

**Figure 7:**
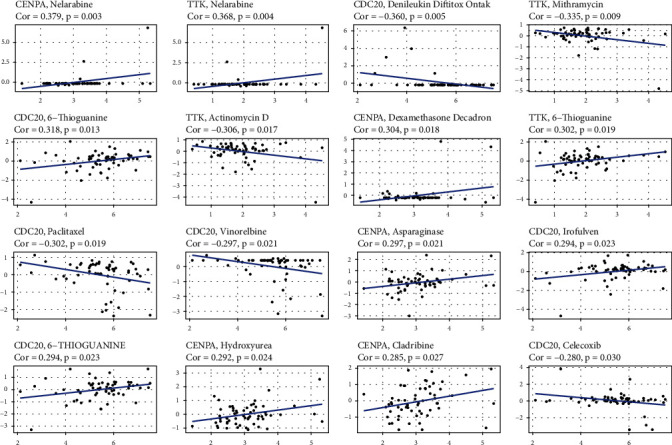
The scatter plot showed the relationship between prognostic gene expression and medication sensitivity.

Gene expression levels and medication sensitivity were examined in NCI-60 cell lines to identify prognostic genes. Several genes were found to correlate with chemotherapy treatment sensitivity (Figure 7). For example, the enhanced expression of CENPA was related to increased treatment resistance to nelarabine, asparaginase, dexamethasone decadron, cladribine, and hydroxyurea. In cancer cells, increased TTK expression was linked to increased resistance to nelarabine, mithramycin, and actinomycin D, 6-thioguanine. CDC20 expression was also associated with higher treatment resistance to denileukin diftitox Ontak, 6-thioguanine, paclitaxel, vinorelbine, irofulven, and celecoxib.

## 4. Discussions

Cirrhosis is the leading cause of death in the liver, and HCC is on the rise [23]. A multidisciplinary approach is required to treat HCC, including hepatologists, surgeons, radiologists, pathologists, and oncologists [24, 25]. Researchers have studied the pathophysiology and epidemiology of HCC for several years. The prognosis for HCC remains dismal, despite substantial advances in surgical and medicinal treatments. This illness develops because early-stage detection methods are lacking [26, 27]. As well as being a very diverse illness, median survival times vary greatly between individuals of comparable TNM stages. In order to tailor prevention and treatment for HCC, it will be crucial to find a powerful prognostic marker that can dynamically reflect the biological progression of the disease [28, 29]. The DDR process affects treatment response and tumor development in patients with HCC. To predict the prognosis of HCC, Li et al. developed a seven-gene signature linked to DNA repair [30]. In order to create a prediction model, genes involved in DDR should be analyzed for their expression patterns.

By analyzing the expression profiles of DDR-related genes in the TCGA database, this study examined the association between DDR-related genes and the prognosis of HCC patients. DDR-related genes were not observed to be differentially expressed between HCC specimens and nontumor specimens at first. Based on the univariate Cox regression analysis, nine DDR-related genes were associated with OS. We also developed the OS-related prediction model, a standalone prognostic indicator for HCC patients, using multivariate Cox regression to identify the three DDR-related genes (CDC20, TTK, and CENPA). As high-throughput sequencing technology and bioinformatics have advanced rapidly, many signals have been developed for predicting prognosis in HCC patients. In contrast to our investigations, these investigations lacked independent validation using external datasets. Additionally, they ignored conventional clinical measures in favor of genetic biomarkers. The study shows promise for therapeutic applications by integrating clinical indicators with the autophagy-related signature to predict survival in HCC patients.

There is evidence that CDC20, TTK, and CENPA were expressed and active in several types of cancer. Zhao et al. demonstrated that knocking down CDC20 improved radiation treatment of growth retardation in HepG2 after radiation activated P53. HCC cells may undergo DNA damage, DNA repair loss, G2/M arrest, and apoptosis when CDC20 is downregulated and radiation is applied [31]. According to Yang et al., CDC20 expression in HCC and HCC cell lines is associated with poor prognosis. Cell proliferation, migration, and invasion of HCC were inhibited by silencing CDC20. Furthermore, silencing CDC20 increased E-cadherin expression while decreasing N-cadherin, vimentin, and Ki-67 expression [32]. A total of 77.63 percent (118/152) of HCC tissues overexpressed TTK, according to Liu et al. [33]. TTK expression and portal vein tumor thrombus presence showed a positive correlation. In HCC, TTK's promoter was demethylated, increasing its expression. Tests in vitro found that TTK improved anchorage-independent proliferation, cell migration, and anchorage independence. Based on the results of the following study, TTK activated the Akt/mTOR pathway in a p53-dependent manner. In several studies, TTK has also been shown to be predictive of HCC. A prior study found that tumor tissues exhibited a marked increase in CENPA mRNA compared to neighboring tissues. In HCC patients, increased CENPA mRNA was associated with elevated alpha-fetoprotein, advanced TNM stage, larger tumor size, advanced AJCC stage, and advanced pathology grade. CENPA, however, was not examined in earlier research. There have been few studies on the roles of CDC20, TTK, and CENPA. This study validated earlier findings that CDC20, TTK, and CENPA are upregulated in HCC. CDC20, TTK, and CENPA prognostic models showed remarkable capability in predicting clinical prognoses for patients with HCC.

According to new research, immune cells in the TME play an important role in cancer development [34]. Among the innate immune cells that can promote or support tumor growth are macrophages, neutrophils, dendritic cells, innate lymphoid cells, myeloid-derived suppressor cells, and natural killer cells [35, 36]. In the TME, cancer cells showed iron ion aggregation during active proliferation. Controlling ferroptosis, therefore, may effectively eliminate tumor cells in terms of iron homeostasis. Besides monitoring tumors and tumor immunity, ferroptosis also has an important immunological function. By combining an examination of distinct immune infiltration densities in the tumor core and the invasive margin, it has been shown that the prognosis of BC patients with poor clinicopathological criteria may be accurately predicted [37, 38]. According to a previous study, the prognosis for patients with HCC is related to the pattern of infiltrating immune cells in TME, and macrophage-associated cytokines may be used to predict PD-L1 levels in these patients [39]. Immune score models based on immune cell infiltration can also predict the prognosis and efficacy of chemotherapy treatment for HCC patients [40, 41]. A study of the prognostic value of the immune infiltration alteration is therefore worthwhile and practical. This study demonstrated that high levels of aDCs, macrophages, Tfh, Th1 cells, and MHC class I were detected in the high-risk group, indicating disruption of immune regulation. Due to this, it may be logical to believe that the antitumor immunity of the high-risk group is weakening, which may explain its poor prognosis.

The drug sensitivity of various anticancer medicines was determined in the treatment of patients with HCC [42, 43]. Data from NCI-60 cell lines showed that higher expression of several prognostic genes was associated with enhanced drug resistance to numerous FDA-approved chemotherapeutic medicines, including denileukin diftitox Ontak, paclitaxel, vinorelbine, and irofulven [44]. Few medicines were also more sensitive to drugs due to a range of prognostic genes. CENPA expression was associated with higher drug resistance to nelarabine, asparaginase, dexamethasone Decadron, cladribine, and hydroxyurea in cancer cells. In order to overcome drug resistance, chemotherapeutic drugs must be tested according to the molecular subtypes of patients.

A range of studies were applied to construct prognostic signatures and numerous verifications using bioinformatics tools and statistical approaches, but there were still some limitations. The samples were provided by a single database, so they may be unrepresentative. Besides, no in vitro or in vivo experiments were conducted. Our future study will focus on the shortcomings listed above.

## 5. Conclusions

A DDR-related signature has been identified as an independent predictor of HCC. A comprehensive analysis of the signature's role in the immune landscape and therapies was conducted. Informing the treatment of HCC with this hallmark could be powerful and promising.

## Data Availability

The data supporting the conclusions of this article will be made available by the authors, without undue reservation.

## Supplementary Materials

Supplementary Materials Figure S1: function analysis of 151 survival-related DDR-related genes. (A) GO enrichment analysis. (B) KEGG analysis.

## References

[1] Villanueva A. (2019). Hepatocellular carcinoma. *The New England Journal of Medicine*.

[2] Mranda G. M., Xiang Z. P., Liu J. J., Wei T., Ding Y. (2022). Advances in prognostic and therapeutic targets for hepatocellular carcinoma and intrahepatic cholangiocarcinoma: the Hippo signaling pathway. *Frontiers in Oncology*.

[3] Komuta M., Yeh M. M. (2020). A review on the update of combined hepatocellular cholangiocarcinoma. *Seminars in Liver Disease*.

[4] Kee K. M., Lu S. N. (2017). Diagnostic efficacy of ultrasound in hepatocellular carcinoma diagnosis. *Expert Review of Gastroenterology & Hepatology*.

[5] Gish R. G. (2006). Hepatocellular carcinoma: overcoming challenges in disease management. *Clinical Gastroenterology and Hepatology: the official clinical practice journal of the American Gastroenterological Association*.

[6] Befeler A. S., Di Bisceglie A. M. (2002). Hepatocellular carcinoma: diagnosis and treatment. *Gastroenterology*.

[7] Gao Q., Shi Y., Wang X., Zhou J., Qiu S., Fan J. (2012). Translational medicine in hepatocellular carcinoma. *Frontiers of Medicine*.

[8] Vilgrain V., Abdel-Rehim M., Sibert A., Ronot M. (2014). Clinical studies in hepatocellular carcinoma. *Future Oncology*.

[9] Bolondi L. (2014). State of the art: hepatocellular carcinoma. *Future Oncology*.

[10] Chatterjee N., Walker G. C. (2017). Mechanisms of DNA damage, repair, and mutagenesis. *Environmental and Molecular Mutagenesis*.

[11] Sinha R. P., Häder D. P. (2002). UV-induced DNA damage and repair: a review. *Photochemical & Photobiological Sciences: Official journal of the European Photochemistry Association and the European Society for Photobiology*.

[12] Santivasi W. L., Xia F. (2014). Ionizing radiation-induced DNA damage, response, and repair. *Antioxidants & Redox Signaling*.

[13] Roos W. P., Thomas A. D., Kaina B. (2016). DNA damage and the balance between survival and death in cancer biology. *Nature Reviews Cancer*.

[14] Goldstein M., Kastan M. B. (2015). The DNA damage response: implications for tumor responses to radiation and chemotherapy. *Annual Review of Medicine*.

[15] Dizdaroglu M., Jaruga P. (2012). Mechanisms of free radical-induced damage to DNA. *Free Radical Research*.

[16] Agarwal A., Panner Selvam M. K., Baskaran S., Cho C. L. (2019). Sperm DNA damage and its impact on male reproductive health: a critical review for clinicians, reproductive professionals and researchers. *Expert Review of Molecular Diagnostics*.

[17] Srinivas U. S., Tan B. W. Q., Vellayappan B. A., Jeyasekharan A. D. (2019). ROS and the DNA damage response in cancer. *Redox Biology*.

[18] Reisländer T., Groelly F. J., Tarsounas M. (2020). DNA damage and cancer immunotherapy: a STING in the tale. *Molecular Cell*.

[19] Wang C., Wang H., Lieftink C. (2020). CDK12 inhibition mediates DNA damage and is synergistic with sorafenib treatment in hepatocellular carcinoma. *Gut*.

[20] Lulli M., Del Coco L., Mello T. (2021). DNA damage response protein CHK2 regulates metabolism in liver cancer. *Cancer Research*.

[21] Zhao Z., He K., Zhang Y. (2021). XRCC2 repairs mitochondrial DNA damage and fuels malignant behavior in hepatocellular carcinoma. *Cancer Letters*.

[22] Gomes A. L., Teijeiro A., Burén S. (2016). Metabolic inflammation-associated IL-17A causes non-alcoholic steatohepatitis and hepatocellular carcinoma. *Cancer Cell*.

[23] Moon A. M., Singal A. G., Tapper E. B. (2020). Contemporary epidemiology of chronic liver disease and cirrhosis. *Clinical Gastroenterology and Hepatology*.

[24] Garrido A., Djouder N. (2021). Cirrhosis: a questioned risk factor for hepatocellular carcinoma. *Trends in Cancer*.

[25] Fattovich G., Stroffolini T., Zagni I., Donato F. (2004). Hepatocellular carcinoma in cirrhosis: incidence and risk factors. *Gastroenterology*.

[26] Sangro B., Sarobe P., Hervás-Stubbs S., Melero I. (2021). Advances in immunotherapy for hepatocellular carcinoma. *Nature Reviews Gastroenterology & Hepatology*.

[27] El Jabbour T., Lagana S. M., Lee H. (2019). Update on hepatocellular carcinoma: pathologists’ review. *World Journal of Gastroenterology*.

[28] Nault J. C., Villanueva A. (2021). Biomarkers for hepatobiliary cancers. *Hepatology (Baltimore, Md)*.

[29] De Stefano F., Chacon E., Turcios L., Marti F., Gedaly R. (2018). Novel biomarkers in hepatocellular carcinoma. *Digestive and Liver Disease: official journal of the Italian Society of Gastroenterology and the Italian Association for the Study of the Liver*.

[30] Li N., Zhao L., Guo C., Liu C., Liu Y. (2019). Identification of a novel DNA repair-related prognostic signature predicting survival of patients with hepatocellular carcinoma. *Cancer Management and Research*.

[31] Zhao S., Zhang Y., Lu X. (2021). CDC20 regulates the cell proliferation and radiosensitivity of P 53 mutant HCC cells through the Bcl-2/Bax pathway. *International Journal of Biological Sciences*.

[32] Yang G., Wang G., Xiong Y. (2021). CDC20 promotes the progression of hepatocellular carcinoma by regulating epithelial-mesenchymal transition. *Molecular Medicine Reports*.

[33] Liu M., Zhang Y., Liao Y. (2015). Evaluation of the antitumor efficacy of RNAi-mediated inhibition of CDC20 and heparanase in an orthotopic liver tumor model. *Cancer Biotherapy & Radiopharmaceuticals*.

[34] Bejarano L., Jordāo M. J. C., Joyce J. A. (2021). Therapeutic targeting of the tumor microenvironment. *Cancer Discovery*.

[35] Chen D., Zhang X., Li Z., Zhu B. (2021). Metabolic regulatory crosstalk between tumor microenvironment and tumor-associated macrophages. *Theranostics*.

[36] Laplane L., Duluc D., Bikfalvi A., Larmonier N., Pradeu T. (2019). Beyond the tumour microenvironment. *International Journal of Cancer*.

[37] Wu T., Dai Y. (2017). Tumor microenvironment and therapeutic response. *Cancer Letters*.

[38] Hinshaw D. C., Shevde L. A. (2019). The tumor microenvironment innately modulates cancer progression. *Cancer Research*.

[39] Donisi C., Puzzoni M., Ziranu P. (2021). Immune checkpoint inhibitors in the treatment of HCC. *Frontiers in Oncology*.

[40] Li H., Li C. W., Li X. (2019). MET inhibitors promote liver tumor evasion of the immune response by stabilizing PDL1. *Gastroenterology*.

[41] Zhang B., Tang B., Gao J., Li J., Kong L., Qin L. (2020). A hypoxia-related signature for clinically predicting diagnosis, prognosis and immune microenvironment of hepatocellular carcinoma patients. *Journal of Translational Medicine*.

[42] Inoue T., Tanaka Y. (2020). Novel biomarkers for the management of chronic hepatitis B. *Clinical and Molecular Hepatology*.

[43] Yildiz G. (2018). Integrated multi-omics data analysis identifying novel drug sensitivity-associated molecular targets of hepatocellular carcinoma cells. *Oncology Letters*.

[44] Shen L., Kondo Y., Ahmed S. (2007). Drug sensitivity prediction by CpG island methylation profile in the NCI-60 cancer cell line panel. *Cancer Research*.

