# Supplementary figures and images for: Risk Predictive Model Based on Three DDR-Related Genes for Predicting Prognosis, Therapeutic Sensitivity, and Tumor Microenvironment in Hepatocellular Carcinoma
Source: J Oncol. 2022 Sep 30;2022:4869732. doi: 10.1155/2022/4869732 (PMC9546689; doi:10.1155/2022/4869732)

A

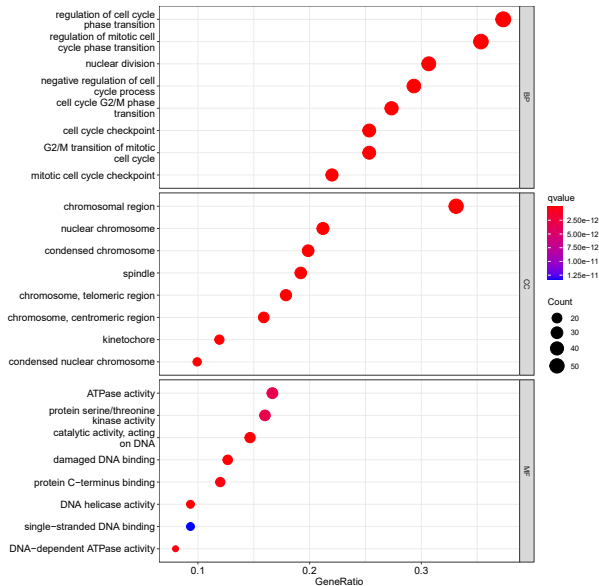

B

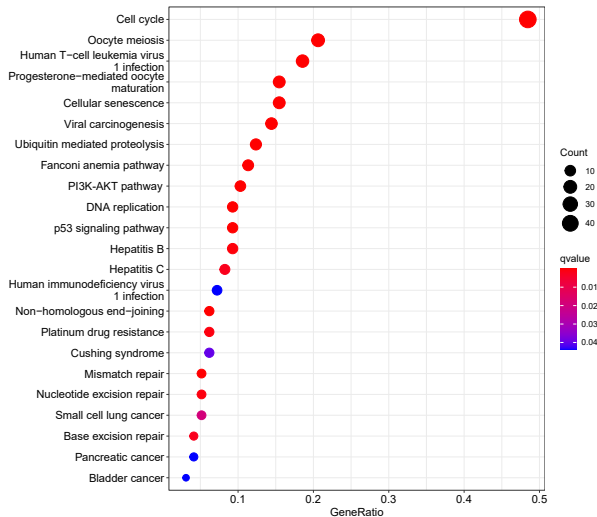

Supplement: Supplementary Materials — Figure S1: function analysis of 151 survival-related DDR-related genes. (A) GO enrichment analysis. (B) KEGG analysis. [file 4869732.f1.pdf]
